# Methodological advances and strategies for high resolution structure determination of cellular protein aggregates

**DOI:** 10.1016/j.jbc.2022.102197

**Published:** 2022-06-24

**Authors:** Anna Schaefer, Dalia Naser, Bruna Siebeneichler, Michael V. Tarasca, Elizabeth M. Meiering

**Affiliations:** Department of Chemistry, University of Waterloo, Waterloo, Ontario, Canada

**Keywords:** protein aggregation, inclusion body, cellular aggregate structure, FTIR, hydrogen–deuterium exchange, NMR, solid state NMR, hydrogen exchange mass spectrometry, Ab, antibody, AD, Alzheimer's disease, AFM, atomic force microscopy, ANS, 1-anilino-8-naphthalene sulfonate, ATR, attenuated total reflectance, CR, Congo red, DNP, dynamic nuclear polarization, EM, electron microscopy, HD, Huntington's disease, IB, inclusion body, MAS, magic angle spinning, MS, mass spectrometry, PD, Parkinson's disease, qHDX, quenched hydrogen deuterium exchange, ssNMR, solid-state NMR, ThT, thioflavin T

## Abstract

Aggregation of proteins is at the nexus of molecular processes crucial to aging, disease, and employing proteins for biotechnology and medical applications. There has been much recent progress in determining the structural features of protein aggregates that form in cells; yet, owing to prevalent heterogeneity in aggregation, many aspects remain obscure and often experimentally intractable to define. Here, we review recent results of structural studies for cell-derived aggregates of normally globular proteins, with a focus on high-resolution methods for their analysis and prediction. Complementary results obtained by solid-state NMR spectroscopy, FTIR spectroscopy and microspectroscopy, cryo-EM, and amide hydrogen/deuterium exchange measured by NMR and mass spectrometry, applied to bacterial inclusion bodies and disease inclusions, are uncovering novel information on in-cell aggregation patterns as well as great diversity in the structural features of useful and aberrant protein aggregates. Using these advances as a guide, this review aims to advise the reader on which combination of approaches may be the most appropriate to apply to their unique system.

Protein aggregation is a widespread phenomenon that is of great importance in disease and in biotechnology. Numerous pathological conditions such as Alzheimer's disease (AD), Parkinson's disease (PD), Huntington's disease (HD), ALS, diabetes, cataracts, and cancer are associated with the deposition of amyloid fibrils and other protein aggregates (1, 2). Moreover, aggregation may occur at many points during the expression, purification, and use of recombinant proteins for medical or biotechnological purposes. Because of their broad significance, great effort has been put into determining the structural and biochemical properties of protein aggregates under native conditions (3). Many of these studies have been performed with hope of selectively preventing or controlling aggregation, while others investigate how protein folding, maturation, and environment can influence toxic aggregates. However, limitations such as structural heterogeneity, sample impurity, and size constraints of structure determination techniques have obstructed a high resolution understanding of protein aggregates.

Historically, protein aggregates were classified into two structural categories: amyloid or amorphous aggregates. By definition, amyloid describes fibrillar aggregates with ordered cross β-sheet structure as identified using electron microscopy (EM) and X-ray diffraction (4). Mature amyloid fibers are often found in the characteristic plaques of protein deposition diseases and thus have been widely studied in terms of their structure, formation, xand toxicity. Commonly, amyloid is formed *via* a nucleation-dependent polymerization. However, many models exist for the exact molecular mechanism (5). In contrast, large, insoluble deposits known as inclusion bodies (IBs) were categorized as amorphous when first discovered (6, 7). IBs commonly form when recombinant proteins are overexpressed in bacteria, but they have also been compared with inclusions found in neurodegenerative diseases such as ALS and HD. For a long time, they were considered deposits of misfolded and inactive proteins that posed an obstacle in recombinant protein production. However, recent studies have revealed that this is not strictly the case and IBs have gained much attention in the fields of biotechnology and medicine as self-immobilized catalysts and drug delivery systems (8).

Over time, descriptions of protein aggregates have become much more detailed and include several other distinct types such as oligomers, protofibrils, and secretory granules, which will all be discussed later in this review. Oligomers encompass a wide variety of small soluble aggregate structures, including protofibrils. Protofibrils may be generally defined as initial transiently populated aggregates that can lead to the formation of mature amyloid; often they are reported to have antiparallel in-register β-sheet character (5). Finally, secretory granules are amyloid-containing assemblies produced by the human endocrine system to sequester hormones for induced release.

Classical techniques for obtaining structural information about protein aggregates typically provide low-resolution data on whether a protein is aggregating and must be coupled with other methods to obtain a more detailed view. One of the simplest approaches to detect the presence of amyloid is UV-visible absorbance or fluorescence spectroscopy to monitor optical changes of the sample upon binding of dye by the aggregate. Commonly used dyes include Congo red (CR), thioflavin T (ThT), and 1-anilino-8-naphthalene sulfonate (ANS). Upon binding amyloid aggregates, CR exhibits characteristic green–yellow birefringence under polarized light and a shift of the absorption maximum from ∼490 nm to ∼540 nm, whereas ThT and ANS exhibit increases in fluorescence when they bind to amyloid species (9, 10). While these dyes have proved useful as diagnostic tools, they are nonspecific and so can bind to other cellular components; for example, binding of CR to certain lipids and ThT to other protein hydrophobic pockets has been described (11, 12). ANS is even more limited because its binding to amyloid structure can induce protein conformational changes (10). Another low-resolution technique is the binding of conformation specific antibodies (Abs), which can qualitatively detect the presence of aggregates and their location. Such Abs have been developed for both sequence dependent and independent (*i.e.*, fibril binding) structure recognition (13, 14). These Abs provide only a slightly more detailed view of structural features or surface exposed residues because they can bind a range of conformations with varying affinities (15, 16, 17).

Other characterization techniques, including protease digestion, atomic force microscopy (AFM), and EM, report on global structure or morphology of aggregates. The degree of digestion with proteases can provide insight on the compactness of the aggregate structure (18, 19), and taking this one step further, fragment sizes can be obtained using subsequent detection by a variety of methods, each providing a different resolution. Approximate fragment sizes can be identified using SDS-PAGE or Western blotting while mass spectrometry (MS) provides much more specific information on what regions of the protein are exposed in the aggregate (20, 21, 22). Similarly, AFM and EM can help visualize macroscopic properties such as length, width, rigidity, and the degree of branching. AFM provides the advantage that it can characterize aggregates at single molecule resolution. An adaptation of transmission EM known as cryo-EM is at the forefront of structural biology; much more detailed three-dimensional structures, with resolutions comparable to X-ray crystallography, can be obtained with the benefits that crystallization is not required, and molecules are not destroyed by the high energy electron beam. These methods will not be considered here as they have been covered in excellent recent reviews (23, 24, 25, 26).

On a microscopic level, secondary structure determination methods such as CD spectroscopy and FTIR spectroscopy can report on aggregates in many physical states (27). While both are able to detect increased β-sheet content, a common feature of aggregating proteins, FTIR provides more detailed observations due to its higher extinction coefficient for β-structures and better fitness for turbid samples. CD has high relative absorbance for α-helical structure, while FTIR provides a more uniform signal for different secondary structures allowing for easier visual assessment of structural changes (28, 29). At the atomic level, methods such as quenched hydrogen deuterium exchange (qHDX) and solid-state NMR (ssNMR) provide high resolution information on which residues are participating in the aggregate structure, allowing for a mechanistic interpretation of aggregate formation (30, 31, 32, 33, 34, 35). These specific methods will be emphasized later in this review as they provide a wealth of complementary and high resolution information.

There is clearly an abundance of techniques to study protein aggregation. Unfortunately, due to limitations caused by sample heterogeneity or impurity, many have largely been limited to studying aggregation *in vitro*—under conditions that may be far different from physiological or pathological conditions. Furthermore, most of the methods applied *in vitro* for the biophysical and structural characterization of these species usually involve purification processes that can perturb the aggregation process and ultimately may change the aggregate structure. Thus, it is crucial that structure determination methods be adapted to study cell-derived aggregates both *ex vivo* and in whole cells or tissue. Here, we discuss the historical basis and recent advances in methods that can be used to study the structure of aggregates, with a specific focus on applications to cell-derived or in-cell and thus impure aggregates; cell-derived will refer to aggregates isolated from human or bacterial cells with minimal processing, whereas purified aggregates will describe purified protein induced to aggregate *in vitro* either by seeding or altered solution conditions. We consider methods from low resolution to high resolution, discuss sample considerations, the strengths and limitations of the methods, and show examples of the types of structural information that can be obtained from each. Determining aggregate structures is of great importance to elucidate critical details of aggregation-prone species and association mechanisms as they apply to disease and biotechnological applications. Thus, we hope this review will serve as an informative guide. Methods reviewed include conformation specific antibodies, attenuated total reflectance (ATR)–FTIR and FTIR microscopy, qHDX with detection by MS or NMR, and ssNMR.

## Conformation specific antibodies: Moving beyond detection to structural information

**Figure 1:**
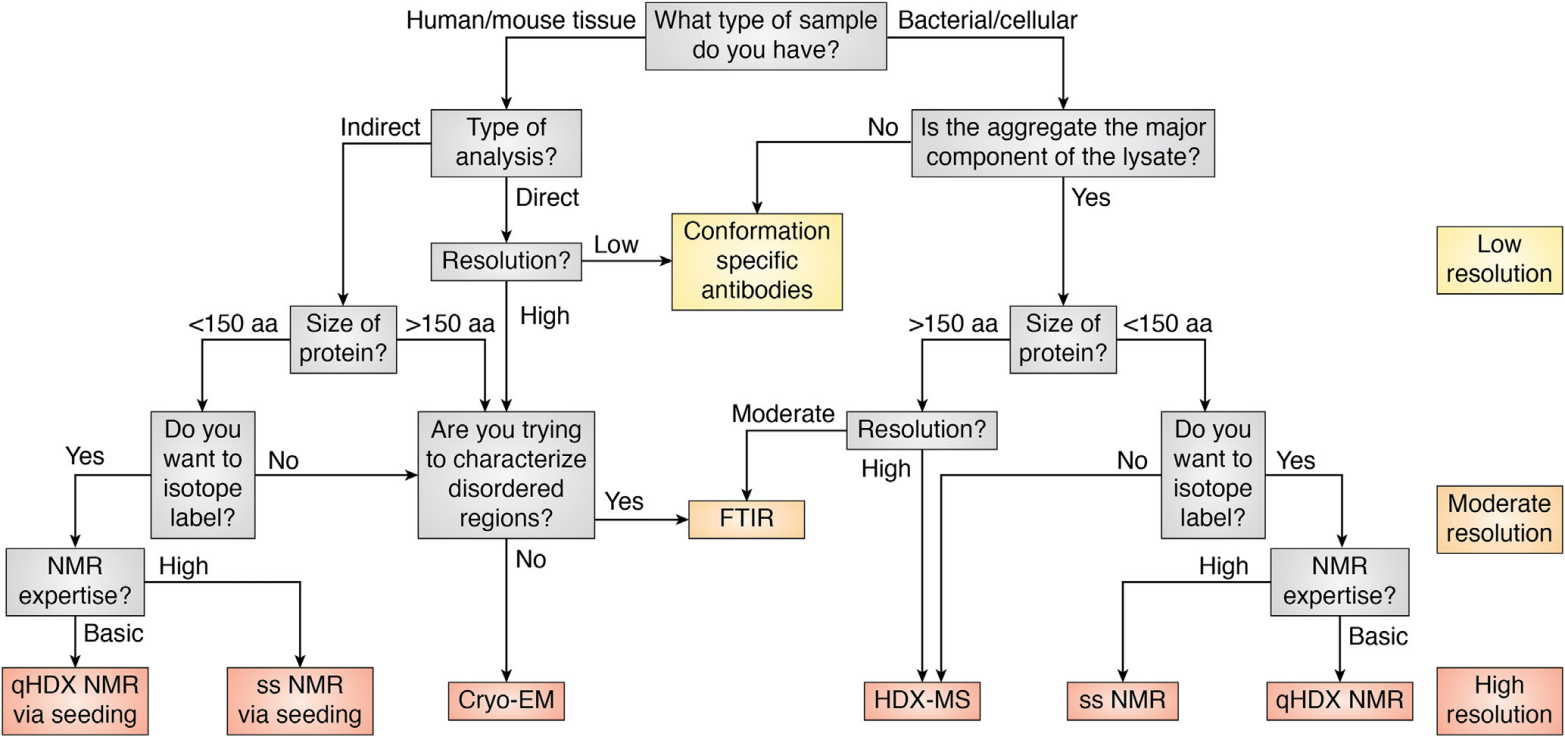
**Technical considerations for the structural characterization of protein aggregates.** Structural characterization methods organized based on resolution, sample limitations such as size and impurity, considerations of time, effort, and cost, and level of expertise.

Antibodies are often used in microbiology methods such as Western blotting, immunohistochemistry, and dot blotting. While these methods do not all necessarily provide structural information, they can serve as a means of determining aggregate composition and a probe for detecting the protein of interest and its localization. The appeal of these methods is twofold: (1) they do not require a large amount of sample and (2) the sample does not need to be pure (Fig. 1). Thus, they are ideal for samples such as lysates (cell or animal) or even patient tissue (36, 37)—to quantify a protein of interest (38) or diagnose a medical condition (39).

Charles Glabe’s group conducted seminal work in using Abs for aggregate structure determination, developing sequence-independent antibodies that recognize amyloid fibrils and oligomers (13, 40, 41). These helpful tools allow discrimination of different types of aggregates and helped broaden the definition of “amyloid” by detecting specific aggregated structures (42, 43, 44, 45). These Abs are now commercially available and have become standard tools to study aggregates. However, further development of Abs has also resulted in protein-, sequence- and conformation-specific Abs against many disease-associated proteins such as Tau, huntingtin, RNA-binding protein FUS, and Cu, Zn superoxide dismutase (SOD1).

For SOD1, Abs have been developed to recognize epitopes throughout the protein structure or detect specific conformations and maturation forms of the protein (46). These Abs can be particularly useful in aggregate characterization. A simple example compared the binding of the B8H10 SOD1 Ab (specific for misfolded SOD1 with exposed loop 4) and C4F6 (specific for oxidized protein) to examine rat spinal cords with mutated SOD1 (14). Pickles *et al.* detected lower binding of C4F6 relative to B8H10, indicating less oxidized protein and increased misfolded SOD1 in the aggregate. They also tested the B8H10 antibody on ALS patient lymphoblasts and saw binding, validating, through indirect structural information, that the mouse model aggregates are structurally like human ones. Conformation-specific and peptide recognizing Abs have also identified distinct conformational strains of aggregates in mutant SOD1 ALS mice models (47, 48, 49). Similar work has been done with Abs against Tau protein, in which antibodies have been shown to bind oligomeric rather than monomeric protein in aggregates, and have identified a key aggregation-prone region in the proteins’ N-terminal domain (50, 51) and on expanded huntingtin polyQ domains, wherein Ab studies discerned multiple toxic conformations (52).

An interesting recent example of the power of Abs to contribute aggregate structural information was provided by Semmler *et al.* (53). Through immunoprecipitation with two SOD1 Abs analyzed by MS, they identified a particular ubiquitin ligase-binding partner associated with mitochondrial misfolded SOD1. This indicated that a specific SOD1 loop is exposed for this interaction to occur. Investigation of other SOD1 mutants *via* Ab binding ascertained that not all variants could interact with the ligase, suggesting the existence of different misfolded structures and misfolding pathways. While this work does not directly investigate the aggregate structure, Semmler *et al.* demonstrate, through discovery of the binding sites of associated proteins, how moderate structural resolution information about exposed segments in the *in vivo* misfolded SOD1 species can be obtained.

Another interesting study by Pickles *et al.* (16) tracked SOD1 aggregation through disease progression by monitoring Ab binding to different rat tissues over time. Through use of Abs that bind throughout the structure, the group established that there were two distinct classes of aggregates: one structure that exists primarily in the motor neurons and neutrophils and a second that exists as a fibrillar network in neurons, axons, and dendrites. Additionally, dot blot experiments on pure SOD1 in different maturation states revealed that the Abs preferentially bind unmetalated (apo) and disulphide reduced protein over more mature folded protein. Other studies on SOD1 Ab affinities have been conducted and have also found preference for specific aggregate structures or different ALS mutant SOD1s (17). Interestingly, it was shown that not all the Abs were reactive in a Western blot, providing a note of caution when interpreting Western blots.

While none of the aforementioned is high resolution structural information, each piece is a structural clue, revealing that distinct structures exist, and potentially the order in which they are formed in a structural maturation process. These types of results highlight the applicability of Abs for in-cell studies, which is especially important as protein folding, maturation, and environmental factors can influence the toxicity of aggregates, and these conditions may be hard to mimic *in vitro*. Thus, many researchers have utilized Abs to investigate toxic species in diseased tissues (15, 16, 40, 46, 54, 55, 56). One example of this strategy used hypersensitive Abs reporting on two known conformations of Aβ aggregates. By time-dependent staining of plaques from mouse tissue, Nyström *et al.* (56) identified a unique structural transition of the plaque core at old age that they proposed could be the cause of a shift in toxicity. Similarly, when examining brain tissue, conformation specific Abs identified amyloids formed by huntingtin exon-1 with looser loops and turns are more toxic than rigid, extended fibrils (55).

In summary, the use of conformation specific Abs has gained traction, with creative researchers applying them in new and exciting ways to obtain structural information on impure and even patient aggregates. Unfortunately, the results are limited by relatively low resolution and because Abs typically bind with varying affinity to different conformations of a protein, limiting precise interpretation. Nevertheless, increasing studies performed with Abs are obtaining disease relevant information including localization and the identification of toxic species in diseased tissue. It is expected that as more conformation-specific Abs continue to become commercially available, there will be increasing applications of these valuable tools in future studies as diagnostic tools and therapeutics for protein aggregation diseases (57). The work done on systems such as SOD1 wherein many Abs have been tested for binding specific structures and sequence illustrates the value of such expansion.

## FTIR: Secondary structure at a glance

FTIR is a rapid and moderate sensitivity means of monitoring protein secondary structure and performing compositional analysis for biological samples. FTIR spectra report on covalent bonds that absorb in the IR region and result in a characteristic fingerprint for a given molecular mixture. Scientists studying protein conformational changes in the context of protein dynamics, misfolding, and aggregation rely specifically on the carbonyl (C=O) stretching vibration of amide bonds, which appears in the 1600 to 1700 cm^−1^ range, termed the amide I region. This range has characteristic frequency ranges for α-helical, β-strands, random coil, loop, and turn structures due to distinctive hydrogen-bonding patterns of the protein backbone, and signal is largely independent of primary sequence allowing facile structure comparison between proteins (27, 58). As extensive FTIR studies have been performed on protein aggregates, a wealth of previous knowledge is available about their spectral characteristics, which can assist current researchers with their investigations (Table 1). Additional signals, termed amide II and III, are observed at 1500 to 1600 cm^−1^ and 1175 to 1310 cm^−1^, respectively. These regions are infrequently used for structure analysis due to their lower intensity and overlap with absorbance bands of amino acid side chains in the case of amide II. However, recent work has highlighted the potential of amide III to be very sensitive to secondary structure and assignments of secondary structure to specific wavenumbers were completed, similar to what has been done for amide I (59).

**Table 1:** FTIR spectroscopy on cellular protein aggregates reveals characteristic spectral features of different β-structures

Bands (cm^−1^)	Assignment	Sample	Technique	Reference
Tissue-derived aggregates				
1620–1618	Amyloid	p53 (breast and lung carcinoma)	FTIR	(152)
↑1627	Inter β-sheet (more amyloid)	Mouse advanced age (AD brain tissue)	μFTIR	(153)
1626	Amyloid	Human AL amyloidosis (heart muscle and heart fat)	ATR-FTIR	(154)
1620/1628/↓1690	Fibrils (parallel β-sheet)	Aβ plaque core (AD brain tissue)	FTIR	(87)
1630/1692	Oligomers (Antiparallel β-sheet)	SOD1 mutant (rat model of ALS)	μFTIR	(80)
Cell-derived aggregates				
1632	Inter β-sheet	Transthyretin exposed cell	μFTIR	(155)
↓1640	↓ Native β-sheet		μFTIR	
↑1620–1635	Amyloid	Aβ (AD nerve cell)	μFTIR	(156)
1625	Inter β-sheet	Ataxin-3 variants (intact cell)	μFTIR	(85)
1642–1640/1630	Native β-sheet (oligomers)			
Cell-derived IBs				
1622	Amorphous aggregates	Asparaginase (induction at 37–42 °C)	ATR-FTIR	(19)
1628–1629	Amyloid aggregates	Asparaginase (induction at 20–30 °C)	ATR-FTIR	
1632	Coiled-coil	ZapB	ATR-FTIR	(71)
1651	α-helix		ATR-FTIR	
1620–1630	Inter β-sheet	Lipase	FTIR	(157)
1650	α-helix			
1680	β-turn			
1623	Aggregates	Phospholipase A2	ATR-FTIR	(158)
1634	Native β-sheet			
1650	α-helix			
1625	Amyloid	VP1-GFP in *Pichia pastoris*	ATR-FTIR	(159)
1680	Antiparallel β-sheet			
*In vitro*-derived aggregates				
1622	Inter β-sheet	SOD1 fibers (60 °C + shaking)	FTIR	(160)
1618	Inter β-sheet	Human Serum Albumin (heated)	ATR-FTIR	(161)
1630/1690	Amyloid (parallel β-sheet)	Aβ42 fibril	ATR-FTIR	(79)
1630/1690	Oligomers (Antiparallel β-sheet)	Aβ42 oligomer	ATR-FTIR	

Details of recent FTIR analyses are compiled ↑ denotes an increase in band signal, ↓ denotes a decrease of band signal.

Abbreviation: μFTIR, FTIR microspectroscopy.

**Figure 2:**
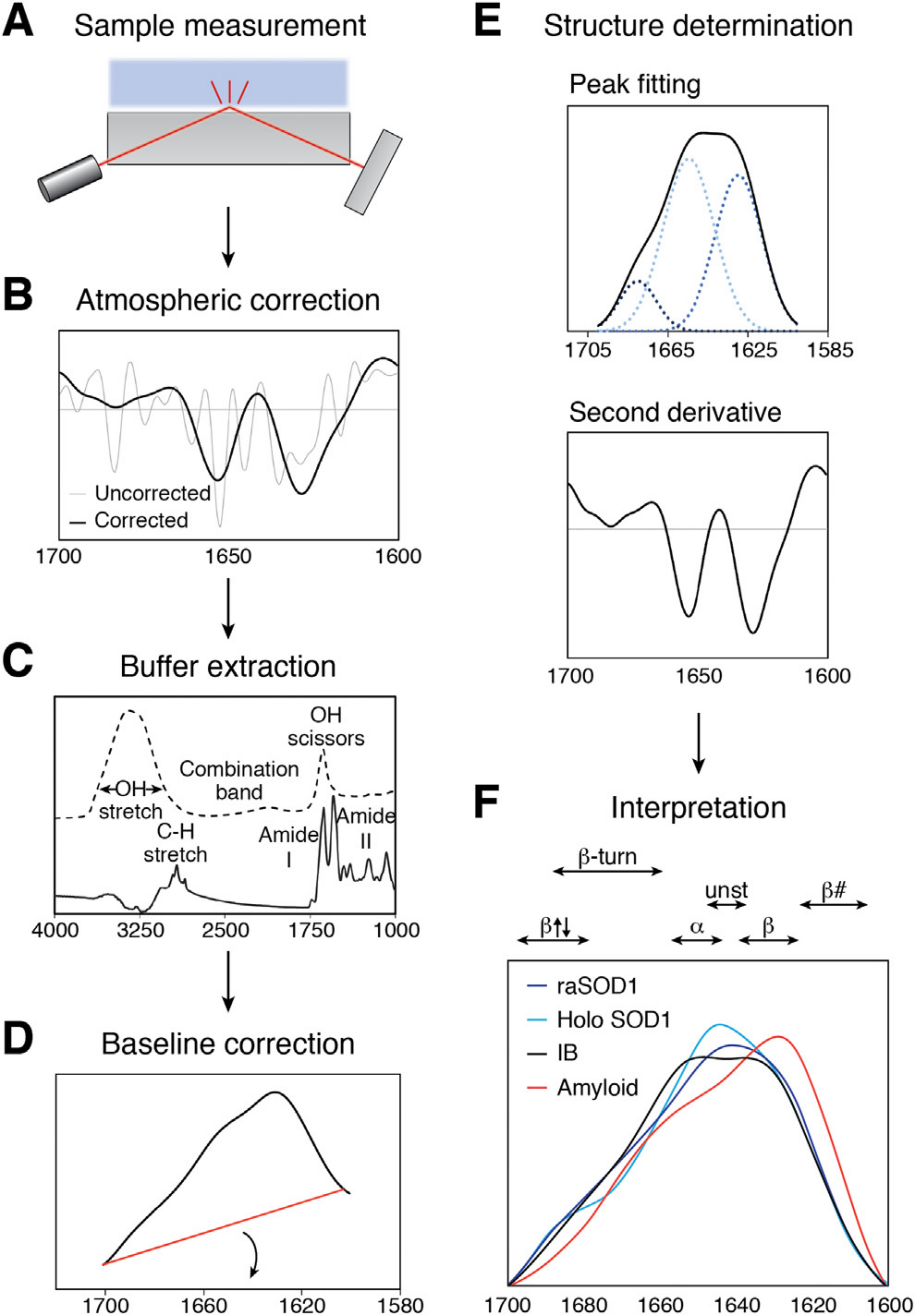
**Data processing workflow for FTIR analysis of protein aggregates.** Following sample measurement (*A*) on ATR-FTIR, data are transformed to give an absorbance spectrum. This absorbance spectrum is sensitive to water and carbon dioxide in the atmosphere and therefore can be corrected using patented algorithms from acquisition and analysis software such as Bruker Opus (*B*). Following atmospheric correction, a post-buffer scan must be subtracted from the sample to remove contributions from water (*C*). A flat baseline is applied to the amide I region (1600–1700 cm^−1^) (*D*) and then the resulting spectrum can be analyzed using structure determination methods such as multivariate peak fitting, second derivative analysis, Fourier self-deconvolution, and more (*E*) (60). Interpretation is often aided by comparison to FTIR spectra of purified proteins. Data are for SOD1, where panel (*F*) is modified from Naser *et al.* (33).

Despite distinct maxima for different secondary structures in the amide regions, the wide bands and small spectral range cause significant overlap and as a result a broad absorbance spectrum. Therefore, for detailed interpretation, additional information must be extracted; common ways to extract secondary structure information include second derivative analysis, Fourier self-deconvolution, multivariate peak fitting, and cluster analysis (60). While the methods noted previously can be combined to provide absolute quantitation, it should be cautioned that peak fitting and Fourier self-deconvolution can be easily biased without proper instruction or expertise. Furthermore, the Savitzky–Golay algorithm is commonly applied during analysis to remove artifacts caused by noise and can also cause the loss of finer details. Therefore, the field generally relies on comparative analyses to avoid incorrect interpretation (61). For a graphical description of FTIR data processing methods, refer to Figure 2.

Protein aggregates have been extensively studied with FTIR, as this method circumvents the typical limitations of structural analysis techniques: it has short sample measurement times allowing the observation of time-dependent changes, it requires relatively low sample amount (<100 ng), and is applicable to insoluble and impure samples (58). Although both transmission and ATR FTIR have been used to study aggregates, ATR-FTIR has become the preferred method as it is more tolerant of insoluble sample components and less sample preparation is required. FTIR spectra of proteins associated with neurodegenerative diseases such as Aβ (Alzheimer’s), α-synuclein (Parkinson’s), and many others have been acquired for fibrils produced *in vitro* (62). These studies revealed a key spectral shift in the β band toward lower wavenumber (1611–1630 cm^−1^) in fibrils, allowing differentiation of native and amyloid-like intermolecular β-sheets (63). Recent studies show a broad spectral range for intermolecular β-sheets; an FTIR study on synthetic amyloid β revealed the size of oligomers correlated with the FTIR absorbance with larger oligomers having lower wavenumbers (64). Others have distinguished between parallel and antiparallel β-sheet shift assignments based on the appearance of a higher order β peak above 1685 cm^−1^ in antiparallel aggregates (65, 66, 67). All of the preceding information cannot be measured by other secondary structure determination techniques such as CD nor by high resolution techniques such as qHDX.

The aforementioned findings from studies of pure protein aggregates allowed enhanced interpretation of *in vivo* aggregation. Many cell-derived aggregates, including bacterial IBs, show increased β character (68, 69), and in some cases, amyloid-like features have been identified in cell-derived IBs using FTIR (68, 70). More recently the coexistence of fibril-like intermolecular β-sheets with significant native structure (19, 33, 71, 72) has further elucidated the heterogeneity of IBs. As the amount of residual native structure in IBs often correlates with the yield of enzymatically active protein, FTIR can be an essential screening tool for researchers trying to understand and optimize functional IBs (72, 73, 74). Similar screening has been performed to investigate the structure of tagged IBs where the tag of choice can facilitate the assembly of the protein of interest into active aggregates (75). Moreover, the effect of expression temperature and culture pH has also been investigated in the context of functional IBs (19, 76, 77, 78). However, conflicting conclusions have been reported for the temperature dependence of IB structure. Studies on lipase from the psychotropic bacterium *Pseudomonas fragi* (PFL) indicate increased native-like structure by FTIR and increased biological activity at lower temperatures (76). Conversely, asparaginase II IBs exhibited increased native structure and higher enzymatic activity at higher temperatures (19). It should be noted that this contradiction may be due to other biochemical properties of the enzymes, such as thermostability, affecting the structural ensemble in the cell and altering which conformations are involved in aggregation. In such studies, for accurate interpretation, the protein of interest should be the main component and not be obscured by cellular background.

Although FTIR has proven useful for the study of purified material and isolated aggregates, the study of whole cells or biological tissues was historically limited by their heterogeneity. Advances in other fields such as optical microscopy and scanning probe microscopy facilitated the development of FTIR microspectroscopy for in-cell studies. Coupling these techniques allows one to selectively obtain IR absorbance readings using knowledge from microscopic imaging; structural changes in regions of interest can be identified without interference from irrelevant cellular background (79). Furthermore, this combination offers the advantage of detecting changes in multiple cellular components simultaneously. A main application of FTIR microspectroscopy is as a diagnostic tool to analyze the structure and composition of diseased tissue samples. Examples include those from patients with AD, PD, ALS, and HD (80, 81, 82, 83, 84). Analyses of these tissue samples often involve assessing the biochemical composition of aggregate/deposit structures by monitoring the lipid or DNA content; similar to proteins, these macromolecules have characteristic FTIR absorbances that can be spatially monitored (83, 85, 86). Lipid content is monitored using the C–H stretching vibration, which appears in the region 3000 to 2800 cm^−1^ and DNA or phosphorylation is monitored using the stretching vibrations of the P-O double bond appearing between 1237 and 1080 cm^−1^ (83). Examples include findings by Bonda *et al.* that in Huntington’s patients, a reduction of unsaturated lipids in the brain matter accompanies inclusion body formation and by Ami *et al.* who identified modifications to lipid membranes, peptidoglycans, and lipopolysaccharide barriers caused by misfolded human Ataxin-3 (83, 85). While not directly providing high resolution structure, this information reports on the state of the cell and other aggregate components that may modulate aggregate structure.

Increasing spatial resolution of FTIR microscopy has provided a more detailed structural view of aggregates. The coupling of AFM with FTIR is commonly referred to as nano-FTIR because a spatial resolution of 10 to 20 nm can be achieved (79). Nano-FTIR allows one to identify tertiary morphology changes of an aggregate and then interpret the secondary structure changes accordingly on time scales relevant to aggregation. In line with previous studies on purified fibrils, Aβ aggregates in diseased brain tissue undergo a structural maturation process from oligomers with antiparallel β character to mature parallel fibrils, which can be detected as an increase in signal at 1630 cm^−1^ with a concomitant decrease at 1693 cm^−1^. This increase in parallel β-sheet content paralleled large-scale structural changes from diffuse to classic cored plaques (87). Similarly, simultaneous structural and morphological changes were identified throughout the *in vitro* aggregation process of the Josephin domain of Ataxin-3 (88). The results of this study disproved the previous notion that aggregation proceeded through misfolded monomers to fibrils and demonstrated the existence of native-like oligomers that were the precursor to fibrillation. As described by Ruggeri *et al.*, these results are novel to FTIR microscopy as the ability to characterize individual species overcomes the large heterogeneity in aggregate sizes/structures during the transition process.

In summary, traditional FTIR has yielded valuable knowledge of aggregate secondary structure by providing information such as the orientation of β strands and the presence of intermolecular β-sheets. FTIR can be applied to samples without isotopic labeling or advanced sample preparation, facilitating its application to cell-derived samples and providing moderate resolution structural data (Fig. 1). Further, the development of different types of FTIR microscopy has allowed the study of whole cells and tissue samples where simultaneous cellular changes induced by misfolded protein or aggregates can be identified (83, 85). However, with all types of FTIR, great care should be taken during the data-processing and analysis stages to avoid misinterpretation. Early popularity of FTIR for analyzing aggregate structure abated in part due to problems with interpretation but has increased again with more in-depth structural knowledge obtained for a wide variety of aggregates providing a firm base for further investigations (28). It is likely that the active development of hardware improvements will allow the examination of tissues with protein concentrations as low as 0.25 mg/ml, and thus, FTIR (micro)spectroscopy may also become a critical tool in early clinical diagnosis of medical conditions, including AD and cancer (86, 89, 90), and advance many studies of cellular protein aggregation.

## ssNMR: A whole-structure view

ssNMR is a powerful tool for high resolution structure determination of cellular protein aggregates. The more conventional solution-state NMR is at best challenging and often impossible for large assemblies such as protein aggregates; even if aggregates can be suspended in solution, their large size causes them to tumble slowly, giving short transverse relaxation times (T2) and the broadening (or even complete disappearance) of their signal (91). ssNMR has emerged as an alternative tool that does not require protein solubility or crystallinity. Thus, it has been used to discern the atomic-level structure of proteins in high molecular weight complexes, both *in vivo* and *in vitro* (92). In the field of protein aggregation, ssNMR has been used to elucidate molecular mechanisms and determine high resolution structures of disease fibrils, IBs, and functional aggregates (34, 93, 94). This section will summarize key advances, methods, and prospects of ssNMR for characterizing cellular aggregates, including disease amyloid proteins, prions, IBs, functional amyloids, and secretory granules.

The implementation of fast magic angle spinning (MAS) and dynamic nuclear polarization (DNP) techniques were major advances for the application of ssNMR to study biomolecules in a native-like environment (95). MAS was an early breakthrough for ssNMR to deal with direction-dependent interactions that occur in a solid sample. Fast sample rotation during data acquisition serves to average out direction dependent or anisotropic interactions as would naturally occur *via* molecular tumbling in solution NMR. This spinning decreases line broadening which, while having the potential to provide information on protein dynamics, generally obscures important structural information. However, the small polarization of ^13^C and ^15^N in MAS ssNMR was still a challenge causing low sensitivity. The more recently developed technique, DNP, provides a means to improve detection sensitivity by transferring the high polarization of unpaired electrons to the nuclei of interest using matched microwave irradiation (91). DNP and MAS ssNMR can be used together and combined with other advanced NMR techniques, such as higher dimensional experiments, assays for structural restraints, proton (^1^H) detection schemes, and nonuniform sampling methods (96).

**Figure 3:**
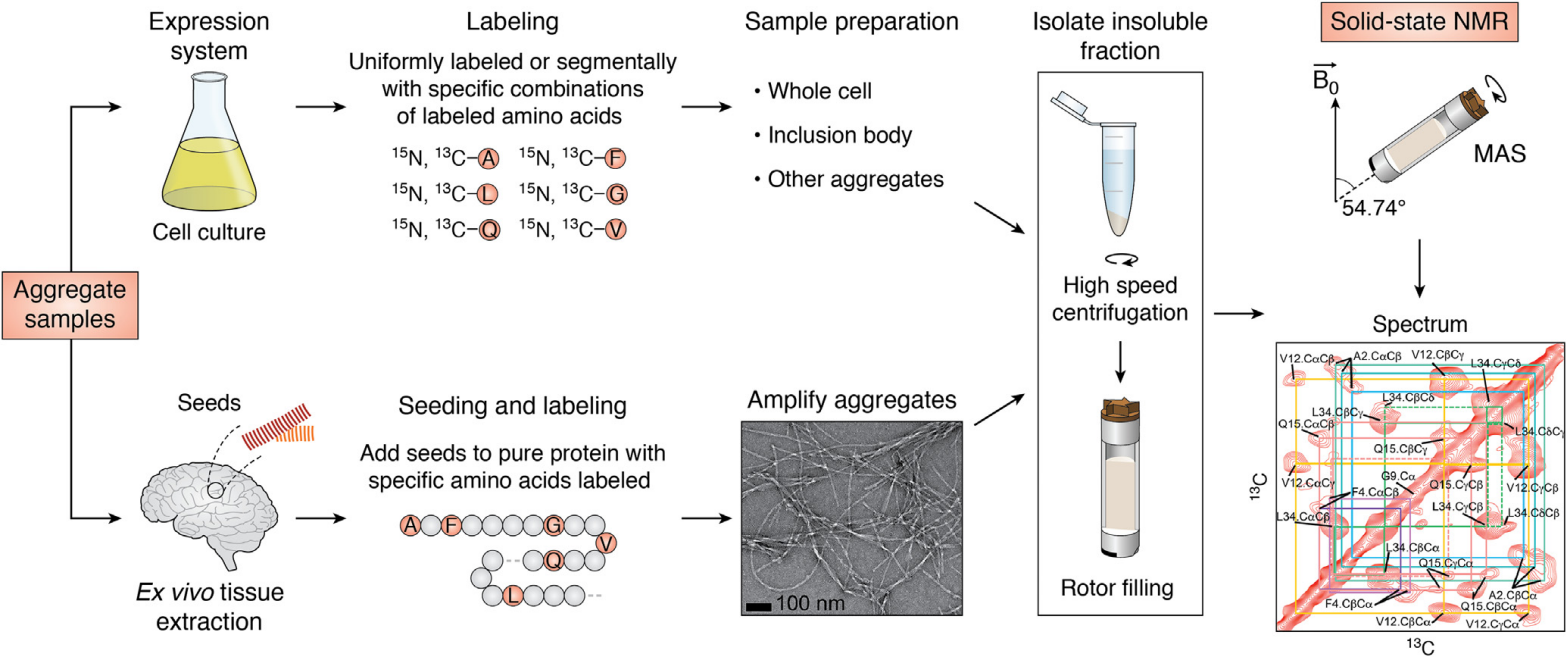
**Sample production for solid-state NMR analysis of protein aggregates.** Aggregates may be obtained from a bacterial cell culture or biological tissue samples. The upper workflow illustrates the incorporation of uniformly or selectively ^15^N, ^13^C-labeled amino acids in aggregates prepared by cell culture. The lower workflow illustrates the preparation of *ex vivo* aggregates where biological aggregates are used as seeds for growing additional aggregate from pure protein, which may contain labeled amino acids (*red circles*). In both cases, the insoluble aggregates are collected by high-speed centrifugation and used to fill the ssNMR rotor. Magic angle spinning is applied to obtain a well-resolved spectrum. In the *bottom panel*, electron microscopy and NMR spectrum for Aβ fibrils are reproduced from Scherpelz *et al.* (97) with permission. ssNMR, solid-state NMR.

ssNMR has been used extensively on amyloid proteins associated with diseases, including AD, PD, HD, systemic amyloidosis, and prion diseases (93, 97). However, these generally have been synthetic or *in vitro* studies (91). Unfortunately, ssNMR experiments on tissue samples are much less common because measurements require milligram scale quantities of isotopically labeled material (Fig. 1). To overcome this limitation, amyloid aggregates isolated from patient tissue (*ex vivo*) with a disease pathology, such as AD, have been used as a source for seeding additional labeled aggregates (Fig. 3) (34). Tycko *et al.* have performed intensive research in Aβ fibrils, illustrating the success of this seeding method (98, 99, 100). Their ssNMR studies on Aβ40 fibrils seeded from various brain regions of Alzheimer's patients revealed that each patient developed a single predominant fibril structure. Moreover, they determined a molecular level structural model for patient Aβ40 fibrils identified distinguishing features from the fibrils produced *in vitro*, a finding that has also been noted by others who elaborate those *in vitro* fibrils generally exhibit a smaller fibril core (101).

Overall, these findings are crucial for the correlation of fibril structures with disease characteristics and hence may assist the future development of AD therapies and diagnostics. Many other amyloids and cellular aggregates formed by proteins such as TAR DNA-binding protein 43 (TDP-43), immunoglobulin light chain, and prion protein (PrP) have also been amplified for ssNMR studies using seeding (102, 103, 104, 105, 106). These studies have been successful in determining residue-specific information on the aggregate structure and describe how broadening of crosspeaks or lack thereof can provide detail on conformational heterogeneity. Technique-wise, they are exemplary of the results that can be obtained from seeding combined with high resolution characterization by ssNMR. Seeding is a valuable tool to obtain sufficient protein for structural studies while retaining structural characteristics of *in vivo* aggregates, allowing more concrete predictions of protein behavior in disease. However, it is important to consider that less abundant or “unseedable” yet still pathologically relevant aggregate species may be missed during the amplification process.

As IBs are often produced during protein overexpression in recombinant bacteria, the sample concentration required for ssNMR studies is more readily achieved. Comparative studies of IBs and fibrils using ssNMR have proven useful in characterizing the composition of IBs for proteins such as the prion-forming domain of HET-s fungal prion (HET-s 218–289), Aβ peptides, and β2-microglobulin (107, 108, 109). The analysis of HET-s (218–289) revealed that both IBs (raw and partially purified) spectra reproduce all the peaks visible for HET-s (218–289) fibrils assembled *in vitro* (107). In contrast, Aβ and β2-microglobulin IBs demonstrated fewer similarities, indicating more heterogeneous ensembles with more contributions from non-β-sheet conformations (108, 109). While the increased protein content of IB samples is advantageous, the heterogeneity of these aggregates can present a challenge. Often IBs exhibit additional resonances arising from phospholipids from the *Escherichia coli* membrane, other proteins, or RNA. Therefore, it is common practice to perform a partial or total purification of IBs using detergent or buffer washes, which may decrease but not eliminate these additional resonances (108). Another approach to reduce the complexity of ssNMR spectra is selective isotopic labeling. An example strategy is the use of specifically ^13^C- and ^15^N-labeled amino acids corresponding to unique residue pairs. This strategy has been employed to obtain residue-specific secondary structure information for aggregates formed by a subunit of the influenza virus hemagglutinin protein (FHA2) and the GP41 protein of HIV in whole cells (110, 111). Upon comparison to known patterns in chemical shifts for amyloid fibrils and their respective soluble forms, both studies concluded that the protein of interest retained large amounts of native structure in IBs.

Different labeling schemes that help decrease background signals and isolate individual protein resonances can also be applied to seeded or purified protein (Fig. 3) to resolve ambiguities for residues of interest (97, 112). Whereas uniformly labeled samples are required for sequence-specific resonance assignments, specifically placed labeled residues give well-resolved spectra that can be used to determine long-range intermolecular distance restraints (113, 114). An example is the ssNMR study of the curli protein, a functional amyloid found in the extracellular matrix of *E. coli* involved in biofilm formation, host adhesion, and invasion (115). Schubeis *et al.* employed segmental labeling of repeat sequences in curli to obtain residue-specific assignments and information about the arrangement of the repeats. The resultant spectra of native curli extracted from biofilm and the lab-expressed protein had high similarity, revealing a well-defined β-solenoid structure. Interestingly, this structure has also been identified by ssNMR in human functional amyloids (secretory granules) of the endocrine system (35). Residue-specific labeling has also been applied to the human functional amyloids of the glucagon hormone. Using sets of scattered labels, Gelenter *et al.* (106) demonstrated an unexpected antiparallel hydrogen bonding pattern throughout glucagon fibrils. All in all, nonuniform isotopic labeling methods for larger complexes can aid in the determination of previously undefined structures and interactions within the complex.

In conclusion, there are currently many ways to acquire valuable information about cellular aggregates using ssNMR, either from a bacterial expression system (116), whole cells (110, 111), or from diseased tissue (98). Although more laborious sample preparation is required, including uniform or selective isotopic labeling (Fig. 1), there are many benefits of ssNMR studies. Unlike other methods reviewed here, ssNMR can provide information on amino acid side chains, conformational heterogeneity through crosspeak linewidths, and the fibril core can be identified at a residue-specific level, giving essential information about aggregation mechanisms and crucial relationships between toxicity and structure. To date, relatively few ssNMR studies have been reported for cell-derived aggregates; thus, this is a likely fruitful area for further research.

## qHDX NMR—residue specific view

qHDX with solution NMR readout is a powerful but also little used method to obtain high resolution structural information on large cellular complexes or aggregates. Traditional HDX provides data on native protein structure and dynamics by measuring the extent of protection for individual protein amides (NHs) in a protein structure against exchange with solvent (D_2_O) over time. However, this method cannot be performed on large assemblies directly as they are not amenable to NMR measurements; instead, protection may be measured using an adapted qHDX method.

**Figure 4:**
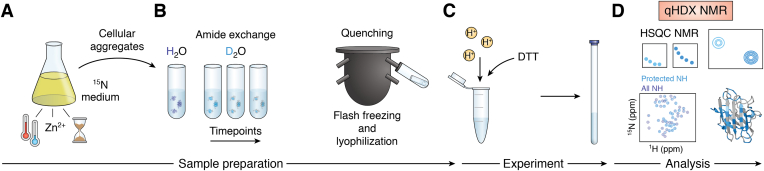
**Quenched amide exchange of a cellular protein aggregate.***A*, samples are grown in ^15^N media; growth variables may include temperature, time, and cofactor addition. *B*, cellular aggregates are then isolated and resuspended in D_2_O and undergo amide exchange for a set time. Exchange is quenched *via* flash freezing and lyophilization. *C*, for measurement, samples are dissolved in a DMSO buffer containing D_2_O, DTT, and DCA to disassemble and unfold the aggregate complex. *D*, the time and temperature of the dissolution is kept constant, and readout is performed using a ^1^H-^15^N-HSQC. Figure modified from Tarasca *et al.* (131). D_2_O, deuterium oxide; DCA, dichloroacetic acid; DMSO, dimethyl sulfoxide; DTT, dithiothreitol.

Upon suspending aggregate samples in D_2_O, NHs that are inaccessible to solvent are protected against exchange, while solvent accessible amides are converted to deuterated amides (Fig. 4). Exchange is then quenched by flash freezing, and the assembly is dissolved in dimethyl sulfoxide, a solvent that slows exchange rates approximately four orders of magnitude compared to aqueous solutions (117). Dimethyl sulfoxide also effectively denatures the protein by lowering the free energy of the unfolded state relative to the native state (118). Thus, the originally too large for traditional ^15^N-based solution NMR assembly becomes an amenable size; the majority of residues may be analyzable for protein subunits smaller than ∼15 kDa. As deuterium is not observed in a ^1^H-^15^N heteronuclear single quantum correlation (HSQC) spectrum, only NHs that were buried or H-bond protected in the assembly, and therefore did not exchange with D_2_O, will be observed. Analysis of HSQC peaks reveals which specific residues were involved in protective structure and which were exposed, providing a high resolution analysis of the protein assembly. The observed protection pattern reports on the average structure of all populated protein conformations in the aggregate. Extensive background on qHDX was described by Hoshino *et al.* (119), and methodological details for samples of pure proteins aggregated *in vitro* were described by Alexandrescu (120).

This method was developed independently by the Alexandrescu (31) and Goto (30) research groups in the early 2000s, and it was initially used to determine structural details of *in vitro* amyloid. Alexandrescu applied qHDX to cold shock protein A (CspA) fibrils that were made through acid denaturing the purified protein. His work elegantly showed that the entirety of the protein was structured in the fibrils, despite their unfolded origins. Soon after, the Goto group reported mapping the core of β2-microglobulin amyloid fibrils. Similar to CspA, all amides, even those natively found in loop regions, showed strong protection against exchange, indicating the whole protein was involved in the fibril structure. In addition, the protection was compared to that of purified native β2-microglobulin and found to be remarkably similar in the core but varied in the loops and termini, suggesting an extensive hydrogen bonding network underlying the rigidity and structural stability of the fibrils. Further application of qHDX to amyloid-β fibrils made from purified protein segments showed, notably, that increasing the length of the aggregating fragment by as little as two residues drastically changed the fibril structure (121, 122, 123).

Some years later, the application of qHDX to cellular IBs was reported by Wang *et al.* in 2008 (32). The cellular IBs were washed several times during their preparation to make largely pure samples. In a remarkable contrast to the preceding studies of *in vitro* aggregates, Wang *et al.* observed protection of only a small proportion of amides in IBs formed by several structurally diverse proteins. Based on the qHDX and complementary experiments, they inferred that the proteins formed small stretches of amyloid and all other residues were unprotected. This result—the first qHDX for an *in vivo* aggregate—showed that cellular aggregation may be very different from *in vitro* aggregation. This was furthered by their 2010 study, where one protein was aggregated in different ways (heat, concentration, acid, amyloid fibrils, and IB) and its structure was analyzed (124). The IB did not resemble the others, further highlighting the importance of *in vivo* studies. Strikingly, the IB resembled the soluble protein more closely than the other aggregates. qHDX studies on washed amyloid-β IBs again showed a new view by revealing the presence of a new, α-helical, aggregation-prone species (108).

**Figure 5:**
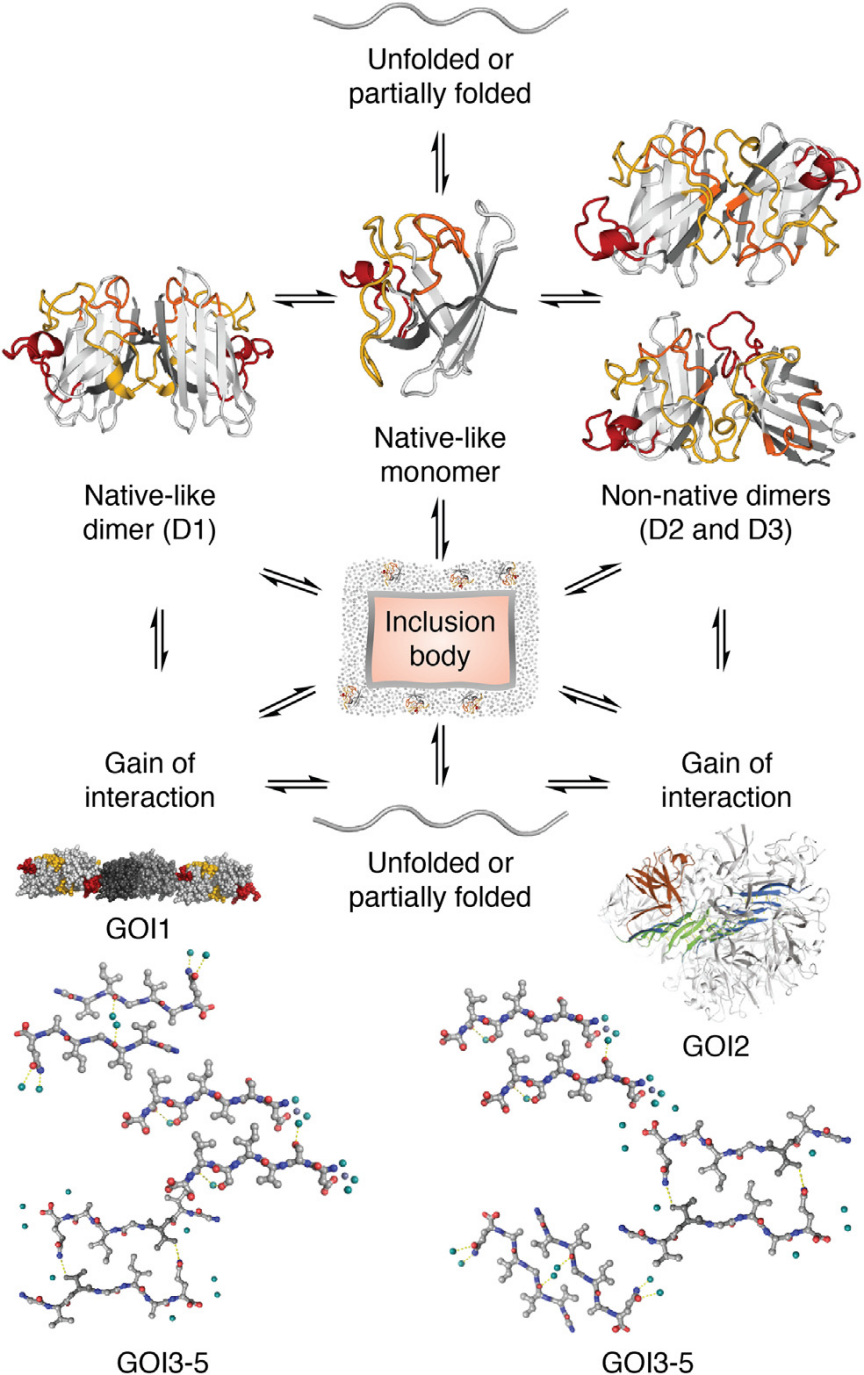
**Ensemble mechanism of SOD1 IB formation.** SOD1 IB formation may arise from multiple pathways that involve native and non-native interactions. The contributions of different pathways can be shifted by mutation. The resulting IB aggregation ensemble includes many structures, which may include D1, D2, and D3 dimer species observed previously by solution NMR and various gains of interaction (GOIs). GOI1 is an “amyloid-like” filament of near-native dimers where L7 (*red*) packs against the β5-β6 edge strands of the next dimer, GOI2 is corkscrew oligomer model, and GOI3-5 are peptide steric zippers. Figure from Naser *et al.* (33). IB, inclusion body.

For some time, qHDX was only used sparingly, predominantly for purified protein aggregates (125, 126), until a recent publication on SOD1 cellular IB structure by Naser *et al.* (33). This study differed from previous work in key aspects. First, the IBs were not washed or purified in any way and incurred minimal processing before amide exchange, thus minimizing the chances of structural alterations. Second, the study was conducted on a diverse set of ALS-associated mutant SOD1s, containing point mutations dispersed throughout the native protein structure and having a wide range of stabilities and other biophysical parameters. Prior to this work, no high resolution study on *in vivo* point mutant aggregates had been reported. Remarkably, almost all the aggregates had indistinguishable structures, despite their native differences. Notably, amide protection was observed throughout the whole protein, corresponding to both natively protected regions as well as loops that are not protected in the native structure. However, some of these loops were shown to be amyloidogenic in predictions and other experiments (127, 128). These results revealed a more complicated picture of aggregation: that the SOD1 IB was an ensemble of different structures that can form *via* multiple aggregation pathways (Fig. 5).

In addition to identifying amide protection pattern, qHDX experiments can be used to study exchange kinetics by measuring protection for different D_2_O exchange times. This allows one to calculate a protection factor and gauge the stability of the aggregate structure (30). It might be expected that this would result in a single exponential decay, as is usually observed for purified proteins. However, while this is observed in some cases (31, 129), a surprising number of the studies show a biphasic or double exponential decay. Yamaguchi *et al.* (130) originally reported that the majority of residues in the β2-microglobulin fibrils could not be well fit using a single exponential function but instead were characterized by a period of fast initial exchange followed by a slower phase or a plateau. This biphasic behavior was explained by a model proposing that the entire fibril was ordered, with the outer edges of the fibrils exchanging quickly. The same residue would also be present in an inner face of the fibril, protected from solvent, resulting in a slower exchange. The combination of these two environments results in biphasic exchange. Wang *et al.* (32) reported similar biphasic behavior for each of several different protein IBs. However, they hypothesized that the fast-exchanging population corresponded to amorphous structure coexisting with slow exchanging, homogeneous, amyloid-like structure. Interestingly, similar biphasic behavior was also observed in qHDX NMR studies of the HypF protein (124), despite the various aggregates in the study having very different structures, suggesting that despite varying aggregation mechanism, in general, aggregated proteins may retain a disordered, amorphous, or perhaps misfolded “shell”. Similarly, fast and slow exchanging populations were also observed in the SOD1 IB studies, suggesting different parts of structures were exposed in a heterogeneous aggregate (33). Unfortunately, qHDX has revealed only the presence and not the structure of this fast-exchanging population, as it is very difficult to monitor and characterize. Nevertheless, the biphasic exchange is an important piece of information that sheds light on why there may be disagreement between results obtained with different structural methods.

qHDX has not yet been applied to visualize aggregation in whole cells and doing so would need to overcome challenges in detecting, identifying, and assigning signals from the aggregated protein of interest. Additionally, parsing the contribution of soluble species of the same protein in the whole cells presents another challenge. On the other hand, the use of qHDX to investigate cell-derived aggregates has many advantages. Because aggregation-prone proteins tend to accumulate in large amounts in cells, relatively small quantities of cell culture can easily be used to generate many NMR samples (131). Combined with the lack of purification minimizing protein loss, this greatly decreases the time and cost for these experiments. Furthermore, the simplicity of the protein preparation and the use of a standard HSQC with minimal setup means that little expertise or specialized equipment is required to obtain significant information on the extent of protection. For more detailed analyses, backbone amide resonance assignment strategies have been documented in detail for easy application, with Tarasca *et al.* demonstrating clearly that point mutants show nearly identical unfolded spectra and that any peaks shifting due to water, pH, or mutation can be easily tracked (131). The low barrier to entry is a significant advantage of qHDX, while residue-specific analysis provides a high resolution view of cellular aggregation that provides a valuable complementary view to results obtained with other methods (Fig. 1).

## qHDX MS: A segment-specific view

**Figure 6:**
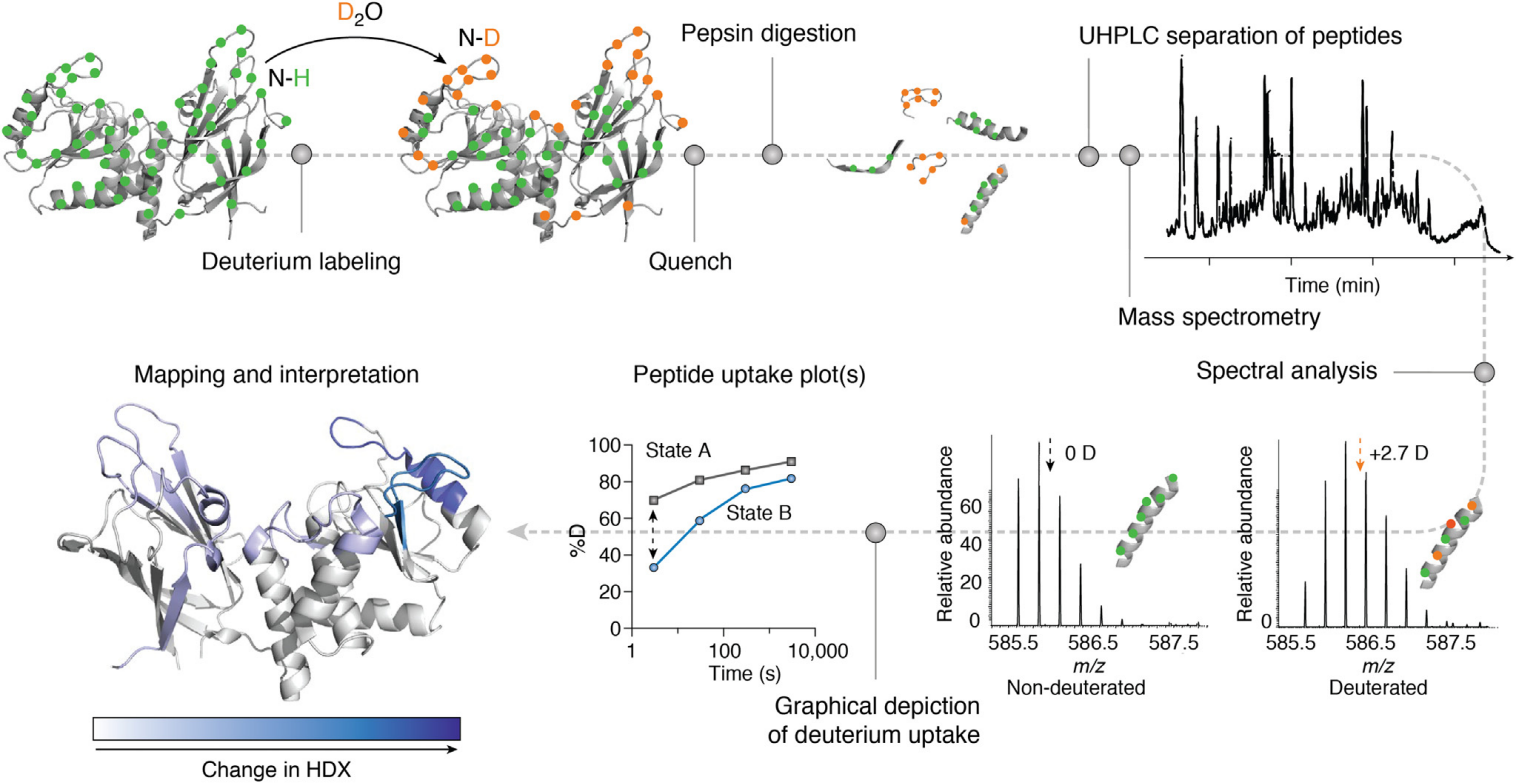
**Overview of a qHDX-MS workflow.** Figure reproduced from Masson *et al.* (134) with permission. MS, mass spectrometry; qHDX, quenched hydrogen deuterium exchange.

An alternative readout to NMR for qHDX experiments is MS, which provides sensitive and precise information on the mass of proteins and peptides. We note that HDX readout by MS or NMR generally provides complementary information, as MS resolves populations that have different net amide protection for a given protein or peptide, while NMR measures the average total protection of individual amides for the entire population (132). In qHDX-MS, exchange in D_2_O is often quenched by decreasing pH, followed by pepsin digestion and MS data acquisition. The extent of deuterium uptake with time can be monitored by MS based on how the mass of each fragment varies; this information can be mapped onto the associated segments of the protein and used to deduce aggregate structure (Fig. 6). This method has been applied extensively to investigate the structure and dynamics of purified proteins; thus there are many existing reviews on its uses (133) and well-developed recommendations on its implementation (134, 135, 136).

Although qHDX-MS is well established, to date it has been little applied to protein aggregates. Furthermore, the studies that have been completed often use purified aggregates to limit the number of additional species in the spectrum that may cause difficulties with assigning fragments of the protein of interest. Interestingly, a recent group of studies on impure PrP structures has provided evidence linking protein structure, infectivity, and function. First, a study on mouse-derived PrP mutant aggregates (PrPsc) revealed evidence for strains, that is, prion proteins with distinct disease phenotypes and distinct structures (137). qHDX-MS showed region-specific differences in deuterium incorporation between three mutants, discerning their different aggregate structures. Additional comparison to amyloid formed by pure PrP also showed clear differences, indicating yet another structure and perhaps an explanation for different infectivities of the aggregates. Further studies on synthetic prion proteins showed differences in deuterium incorporation, distinctive in some helical portions of the proteins (138). These studies also revealed a potential drawback of qHDX-MS when a fragment of the C terminus exchanged very rapidly and then plateaued. This indicates that part of the segment is completely unordered/unprotected, whereas the rest is highly protected; however, it is impossible with this method to tell which residues show which behavior. Thus, while region-specific structural information can be obtained by qHDX-MS, residue-specific interpretation may be limited.

Similar to backbone amides, the imidazole protons of histidine residues are susceptible to exchange with solvent. Therefore, an avenue to obtain residue-specific information is through histidine HDX-MS. By exploiting the differing pH dependence of amide and imidazole exchange reactions, one can distinguish between their protection in an aggregate structure. A study on the human PrP used the combined results of qHDX of imidazole proteins and backbone amides to evaluate the structural differences between recombinant protein and protein seeded with sporadic Creutzfeldt–Jakob disease prions (139). These results provided a good basis for using seeded protein as a more accurate model of disease conditions and highlighted possible differences in *in vivo* aggregation mechanisms.

More recently, Kaldmae *et al.* (140) have attempted to expand the scope of qHDX-MS with the novel implementation of a cellular approach. In this process, cells overexpressing the protein of interest (the N-terminal domain of major ampullate spidroin 1, NT) were incubated directly in a deuterated buffer before quenching, lysing, and performing MS. Deuteration for different amounts of time showed a clear time-dependent mass increase with time, which could be localized to distinct segments of the protein through electron-transfer dissociation sequencing. These results demonstrated that despite extensive aggregation-prone regions in NT, the protein under cytosolic conditions adopts a very tight fold rather than amyloid. This approach was then taken one step further, to test the conformational stability of cellular NT. In-cell deuteration of an Aβ-NT fusion followed by MS detection revealed fibrillar aggregates. However, these fibrils largely did not incorporate NT, indicating it remains in a stable fold under cellular conditions. This phenomenal study clearly highlights the importance of studying protein folding and aggregation *in vivo*, as purified protein did not behave the same way as the in-cell aggregates. Furthermore, in this scenario qHDX-MS was able to provide crucial structural and mechanistic information that would have otherwise remained obscure. While this is just the first example of an in-cell qHDX-MS study of aggregating protein, future developments to ion separation techniques may enable the separation of species based not only on mass and charge but also on shape. Further applications of these exciting qHDX-MS methods are a promising avenue for resolving specific aggregate conformations in living cells (133).

Overall, detection of hydrogen exchange *via* MS provides valuable ways to obtain high resolution information on aggregate structures. Recent advances have allowed for a more in-depth study of in-cell aggregation mechanisms with the advantage that samples do not need to be labeled or purified. Additionally, the amount of sample required for MS is minuscule and the experiment time is short, allowing for technical and biological replicates to be performed more easily than for NMR methods (134). Since *in vivo* conditions are less controlled than *in vitro* experiments, the ability to perform replicates is crucial. However, it should be noted that the presence of increased cellular contaminants will result in a more complex mass spectrum. Therefore, it is important that the protein of interest is present in a reasonably high concentration and/or a purified protein is available as a comparison during fragment assignment (Fig. 1).

## Conclusions

In conclusion, the determination of *in vivo* aggregate structures and mechanisms of aggregation is of central importance to biotechnology and medicine. Amyloid and many other aggregate structures are present in the protein deposits of many maladies, such as AD and PD, ALS, and cancer. Amyloid fibrils have also been identified in functional aggregates in the *E. coli* extracellular matrix involved in biofilm formation and secretory granules of the human endocrine system that work as long-term deposits of hormone units (8, 113). Furthermore, it has been shown that bacterial IBs are composed of functional native and native-like protein structures along with amyloid or aggregated forms of the same protein. This molecular architecture allows for retention of biological activity as well as high mechanical stability, showing promise for their use as functional materials (8, 33). Consequently, IBs are being explored for applications as drug delivery systems and self-immobilized catalysts. IBs formed by enzymes, such as reductases, kinases, lipases, and synthases, have the advantage of catalyzing reactions while being reusable and recyclable, resistant to proteases, and relatively easy to purify (8, 141, 142, 143). In all these occurrences of aggregation, structural and mechanistic knowledge would be an asset, contributing to the development of disease treatment, biotherapeutics, and catalysts in industrial processes, for example (144, 145, 146).

As specifically highlighted in each section, the techniques reviewed here have all advanced to provide crucial structural information on cell-derived and in-cell protein aggregates. Although each has its advantages as a standalone technique, it is common practice to employ multiple techniques to gain an in-depth characterization of protein aggregates. Some methods, such as conformation-specific Abs, capture the surface properties of the aggregate, while others, like FTIR, acquire signals from all components of the aggregate. Some techniques, such as cryo-EM and ssNMR, report mainly on ordered portions of the structure. However, unlike cryo-EM, ssNMR can provide indirect information on residues exhibiting structural polymorphisms through peak broadening. Finally, qHDX with detection by either NMR or MS provides measurement of the aggregate ensemble at atomic resolution. To date, only a small number of investigations of cellular aggregate structures have used methods such as qHDX-MS and NMR. However, recent advances in these techniques have provided exciting prospects for gaining additional groundbreaking insights on cell-derived and *in vivo* aggregates. Similar to qHDX, various complementary MS and NMR methods to study aggregated protein *in vitro* may be adaptable to study cellular or cell-derived aggregates, in a variety of cellular contexts, such as the cytoplasm, cellular compartments, or on membranes, and are promising areas for future research (132, 147, 148, 149, 150, 151). Our critical analysis of methodologies with consideration of sample characteristics such as purity, feasibility of isotopic labeling, concentration or amount, protein size, and level of expertise may serve as a valuable resource for further advancing high resolution knowledge of cellular aggregate structures.

While the aforementioned methods have enabled much progress, overcoming previous limitations such as aggregate size, sample heterogeneity, and insolubility, there are still obstacles to further advances. For the goal of determining aggregation mechanisms and structures in living cells, there is the complication of selectively observing the protein of interest. This means signals from contaminating proteins must be removed or otherwise taken into account. This may be accomplished by analyzing cell-derived aggregates *ex vivo* and can be further aided by sample processing such as washing with buffer or selective solubilization using detergents. Unfortunately, the effects on aggregate structures of such processing and other sample preparation requirements are variable and often unknown. It is essential to consider the consequences of such experimental variables in the application and further development of the structure analysis tools described herein. The current powerful suite of structure analysis techniques, and likely continued developments thereof, are applicable to a great variety of systems. These methods provide a strong foundation for exciting further progress in understanding and ultimately controlling protein aggregation in medical and biotechnology settings.

## Conflict of interest

The authors declare that they have no conflicts of interest with the contents of this article.
